# Carbapenem resistance genes and biofilm−virulence uncoupling in *Acinetobacter baumannii*

**DOI:** 10.3389/frabi.2026.1888133

**Published:** 2026-07-29

**Authors:** Shyam Tripathi, Kusum Rani, Rivan Berlia, Amit Sharma, Anandu Hari, Pratibha Gaur, Poornima Sheba Samuel Raj, V. Samuel Raj

**Affiliations:** 1 Department of Biotechnology and Microbiology, School of Science and Humanities, SRM University, Sonepat, Haryana, India; 2 Centre for Drug Design Discovery and Development (C4D), SRM University, Sonepat, Haryana, India; 3 Department of Infection Prevention and Epidemiology, Amrita School of Medicine, Amrita Vishwa Vidyapeetham, Faridabad, Haryana, India; 4 PRIMSR Hospital, SRM University, Sonipat, Haryana, India

**Keywords:** *Acinetobacter baumannii*, biofilm, blaOXA-23, carbapenem resistance, genotype-phenotype concordance, virulence genes

## Abstract

**Introduction:**

*Acinetobacter baumannii*, a World Health Organization (WHO) critical-priority pathogen, causes difficult-to-treat nosocomial infections. The link between resistance genes, biofilm, and virulence carriage remains poorly understood in clinical isolates of high−burden South Asian settings. This study intended to thoroughly describe and explain linkages among clinical isolates collected in Haryana, India.

**Methods:**

A total of 200 non-duplicate *A. baumannii* isolates underwent antimicrobial susceptibility testing by broth microdilution against 17 antibiotics, interpreted according to CLSI M100 guidelines (32nd edition). Biofilm forming capacity was quantified using the standardized microtiter plate crystal violet assay. Polymerase chain reaction (PCR) was used to detect six carbapenemase genes (blaOXA-23, blaOXA-24, blaOXA-58, blaNDM-1, blaVIM, and blaIMP), beta-lactamase genes (blaPER-1, AmpC, and blaTEM), and virulence genes (bap, ompA, and csuE). Concordance was assessed by Cohen’s κ; gene-resistance and gene-biofilm links were tested using regression and non−parametric analyses. A total of 200 pure, non−duplicate *A. baumannii* isolates were included and contaminated cultures were excluded. Sample size adequacy was assessed using Buderer’s formula based on expected sensitivity, specificity, carbapenem-resistance prevalence, 95% confidence level, and desired precision.

**Results:**

Among 200 isolates, 54% were MDR, 34% XDR, and 61% carbapenem-resistant. The blaOXA-23 gene, present in 61%, showed near-perfect concordance with carbapenem resistance (κ = 0.81). Composite carbapenemase carriage improved concordance (κ = 0.88). Biofilm capacity was unrelated to overall resistance, but strong biofilm formers had higher colistin MICs (p = 0.001). Virulence genes (*bap*, *ompA*, and *csuE*) were linked to strong biofilm formation (p < 0.001) without resistance escalation.

**Conclusions:**

blaOXA−23 emerged as the dominant genetic driver of carbapenem resistance, underscoring its value in rapid diagnostics. Colistin tolerance was linked to biofilm−associated phenotypes rather than cumulative resistance.

## 1 Introduction

*Acinetobacter baumannii* (*A. baumannii*) is a Gram-negative coccobacillus that has evolved into a major ICU pathogen worldwide. *A. baumannii* is listed by WHO as a critical-priority pathogen in both the 2017 and 2024 bacterial priority pathogen lists (Tacconelli et al., 2018; WHO, 2024). Carbapenem-resistant *A. baumannii* (CRAB) causes severe infections including ventilator-associated pneumonia (VAP), bloodstream infections, urinary tract infections, burn wound, and pressure ulcer infections, with resistance rates exceeding 80% in South Asia and ~40% in Europe (Burcu et al., 2018; Antimicrobial Resistance Collaborators, 2022; Najafabadi and Soltani, 2024; Thacharodi et al., 2024).

The antimicrobial resistance (AMR) arsenal of *A. baumannii* is formidable and multifactorial. Enzymatic resistance is dominated by the Ambler class D oxacillinase OXA-23-like carbapenemase, which is frequently mobilized by insertion sequence ISAba1, embedded in resistance genomic islands on conjugative plasmids, and disseminated globally within international clonal complex 2 (CC2) lineages (Thacharodi et al., 2024). Other mechanisms include metallo−β−lactamases, outer membrane protein loss, and efflux pump overexpression (Kyriakidis et al., 2021). This resistance arsenal does not allow many treatment options, with polymyxins and tigecycline increasingly compromised (Mohapatra et al., 2021).

In addition to drug resistance, *A. baumannii* is widely known for biofilm formation, allowing survival on hospital surfaces and devices (Gedefie et al., 2021). For biofilm formation, biofilm-associated protein (Bap), which mediates intercellular adhesion and maturation, outer membrane protein A (OmpA), which drives initial adhesion to epithelial cells and abiotic surfaces, and the chaperone-usher pili (Csu) system, which mediates irreversible surface attachment, are crucial. These determinants collectively enable *A. baumannii* to persist on hospital surfaces, colonize indwelling devices, and resist routine decontamination (Brossard and Campagnari, 2012).

The relationship between biofilm formation and antimicrobial resistance is widely assumed but poorly validated. The “biofilm-resistance coupling” hypothesis and “biological cost” model argues that whether biofilm formation enhances resistance through gene transfer or imposes a biological trade−off remains unresolved (Michaelis and Grohmann, 2023; Pompilio and Di Bonaventura, 2026). Clinically, biofilm-mediated *tolerance* (a reversible, non-heritable phenotypic state) must also be distinguished from conventional genetic *resistance*—a conceptual distinction with direct implications for MIC-based clinical decision-making (Uruén et al., 2020).

Despite the clinical urgency, few studies have simultaneously evaluated resistance genotypes, resistance phenotypes, biofilm capacity, and virulence gene carriage in large, diverse *A. baumannii* collections from high-prevalence South Asian settings, where clinical outcomes are most severely impacted. The gap in knowledge specifically concerns the predictive accuracy of individual carbapenemase genes for phenotypic resistance and the independence (or otherwise) of the biofilm and resistance adaptive axes. This study therefore pursued four explicitly defined objectives: (i) to evaluate the association between biofilm formation capacity and cumulative antimicrobial resistance burden; (ii) to determine the predictive accuracy of *blaOXA-23* and other carbapenemase genes for carbapenem resistance phenotype; (iii) to quantify genotype–phenotype concordance via Cohen’s κ statistics; and (iv) to characterize the functional triangle linking virulence genes, biofilm formation, and resistance profiles. We studied 200 isolates to test resistance-biofilm linkages and identify diagnostic markers.

## 2 Materials and methods

### 2.1 Study design, setting, and ethical approval

The present study is a retrospective, cross-sectional molecular epidemiology study involving archived *A. baumannii* isolates procured from PRIMSR Hospital, Haryana, Delhi-NCR. The clinical isolates were collected from the microbiological laboratories of the hospital network in Delhi-NCR.

The study was reviewed and approved by the Institutional Ethics Committee of SRM University, Delhi-NCR, Sonepat, Haryana, India (Approval No. SRMUH/UEC/2026/001, dated 23 June 2026). The requirement for individual informed consent was waived because archived, de-identified bacterial isolates were used, with no direct patient recruitment, intervention, or identifiable patient information involved.

This approval was granted following prior clearance from the Review Committee on Genetic Manipulation (RCGM), Department of Biotechnology, Government of India, under the Institutional Biosafety Committee (IBSC) meeting held on 24 June 2025 (Registration No. SRMU020520243282).

### 2.2 Bacterial isolates, colony selection, purity check, and species confirmation

A total of 200 non-duplicate *Acinetobacter baumannii* isolates collected between 2022 and 2024 were included in this study. The isolates were obtained from clinical specimens, including blood, broncho-alveolar lavage fluid (BALF), cerebrospinal fluid (CSF), sputum, urine, pus, and rectovaginal swabs, as well as environmental sources, including surface swabs from ward surfaces and medical equipment. Environmental isolates were obtained from routine hospital environmental surveillance and were not specifically collected immediately after a documented environmental contamination episode.

Inclusion criteria were (i) confirmed *A. baumannii* isolates; (ii) non-duplicate isolates, defined as one isolate per patient or one isolate per environmental sampling site and (iii) availability of a pure viable culture for antimicrobial susceptibility testing, biofilm assay, and molecular analysis. Contaminated cultures, mixed cultures, duplicate isolates from the same patient or environmental site, and isolates that could not be revived from storage were excluded.

For each isolate, a single well-separated colony was selected from the primary culture plate within 48–72 h of routine diagnostic isolation and subcultured on fresh MacConkey agar and blood agar to obtain a pure culture. Purity was confirmed by uniform colony morphology, Gram staining, and biochemical profiling before further testing. Cultures showing mixed colony morphology, inconsistent Gram-stain findings, or evidence of contamination were reisolated; isolates that failed purity confirmation were excluded.

Genus-level phenotypic characterization was performed by observing colony morphology on MacConkey agar, Gram staining, and biochemical profiling. Presumptive *A. baumannii* isolates were identified as pale, non-lactose-fermenting colonies on MacConkey agar, Gram-negative coccobacilli on Gram staining, oxidase-negative, catalase-positive, non-fermentative, and non-motile organisms. Species confirmation was performed using conventional microbiological characterization supported by PCR detection of the intrinsic blaOXA-51-like gene (J. F. et al., 2006).

Confirmed isolates were preserved in sterile cryovials containing 20% glycerol and 80% tryptic soy broth (TSB; HiMedia, Cat. M011) at −80 °C until further use.

### 2.3 Antimicrobial susceptibility testing

Antimicrobial susceptibility testing (AST) was determined by the broth microdilution method in accordance with CLSI M100 guidelines (CLSI, 2015; Rani et al., 2024), for 17 antibiotics. Testing was performed in cation−adjusted Mueller−Hinton II broth (BD Difco/BBL™, Cat. 212322). Overnight-grown fresh cultures were used to prepare a 0.5 McFarland bacterial suspension (approx. 1.5 × 10^8^ CFU/mL), as described in our prior publications (Rani et al., 2024; Sharma et al., 2025). Inoculum was prepared by diluting the suspension of 1:100 (~10^6^ CFU/mL) and dispensed into 96-well microtiter plates containing serial 10-fold dilutions of the selected antimicrobial agents. Plates were left to incubate in ambient temperature at 37°C for 16–18 h.

The minimum inhibitory concentration (MIC) was defined as the lowest concentration of each antimicrobial that inhibited visible growth after incubation. Each isolate was tested by broth microdilution, and isolates showing borderline MIC values near clinical breakpoints, unclear growth endpoints, or genotype–phenotype discordance were retested independently from a freshly prepared culture. Replicate MIC results were considered acceptable when values were within ±1 two-fold dilution. In cases of discordant categorical interpretation, the assay was repeated, and the reproducible MIC was used for final analysis. MIC interpretation was performed based on definitions provided in the CLSI guidelines (CLSI, 2015; Rani et al., 2024), whereas colistin susceptibility was determined according to EUCAST recommendations (European Committee on Antimicrobial Susceptibility Testing, 2026). MIC values are presented as median log_2_ MIC with interquartile range because MIC distributions were non-normally distributed.

Antibiotic classes tested included carbapenems (meropenem, Sigma-Aldrich, Cat. No. M2574; imipenem, Sigma-Aldrich, Cat. No. I0160), fluoroquinolones (ciprofloxacin, Sigma-Aldrich, Cat. No. 17850; levofloxacin, Sigma-Aldrich, Cat. No. 28266), aminoglycosides (amikacin, Sigma-Aldrich, Cat. No. A0365900; gentamicin, Sigma-Aldrich, Cat. No. G1264), third-generation cephalosporins (ceftazidime, Sigma-Aldrich, Cat. No. C3809; cefotaxime, Sigma-Aldrich, Cat. No. C7039; ceftriaxone, Sigma-Aldrich, Cat. No. C5793), a β-lactam/β-lactamase inhibitor combination (piperacillin–tazobactam, Sigma-Aldrich, Cat. No. P8396), glycylcycline (tigecycline, Sigma-Aldrich, Cat. No. PZ0021), and polymyxin (colistin sulfate, Sigma-Aldrich, Cat. No. C4461). All MIC values were log_2_-transformed prior to statistical analysis. Resistance categories (MDR, XDR, PDR) were defined as per Magiorakos et al. (2012). Quality control was performed in each AST run using *Escherichia coli* ATCC 25922, *Pseudomonas aeruginosa* ATCC 27853, and *A. baumannii* ATCC 19606. AST plates were accepted only when the MIC values of quality-control strains fell within the recommended acceptable ranges.

### 2.4 Biofilm formation assay

Biofilm formation capacity was quantified by the standardized microtiter plate crystal violet assay as per our previous work with few modifications (Sharma et al., 2025). Briefly, overnight cultures (18 h at 37 °C, shaking at 120 rpm) were adjusted to 0.5 McFarland standard (~1.5 × 10^8^ CFU/mL) in tryptic soy broth (TSB; HiMedia, Cat. No. M011) supplemented with 1% (w/v) D-glucose and inoculated (200 μL per well) into 96-well flat-bottom polystyrene plates (Costar 3599, Corning, USA; Cat. No. 3599). Plates were incubated statically at 37 °C for 24 h.

After incubation, planktonic cells were carefully aspirated, and wells were washed three times with phosphate-buffered saline (PBS, pH 7.4) to remove non-adherent cells. The attached biofilm was fixed with 99% methanol (Sigma-Aldrich, Cat. No. 34860) for 20 min and stained with 0.1% (w/v) crystal violet (Sigma-Aldrich, Cat. No. 61135) for 20 min. Excess stain was removed by washing, and the plates were air-dried. Bound crystal violet was solubilized using 95% ethanol (Sigma-Aldrich, Cat. No. E7148), and absorbance was measured at 570 nm using the Multiskan FC Microplate Photometer.

Each isolate was tested in triplicate, and uninoculated wells served as negative controls for calculating the optical density cutoff value (ODc). The mean OD570 of triplicate wells was used for classification. Assays were repeated if contamination, technical error, or inconsistent replicate readings were observed.

Biofilm producers were classified as non-producer (OD ≤ OD_n_), weak (OD_n_ < OD ≤ 2×OD_n_), moderate (2×OD_n_ < OD ≤ 4×OD_n_), and strong (OD > 4×OD_n_). Growth-curve analysis was not performed in parallel; however, all inocula, media, incubation conditions, and OD-based classification criteria were standardized to minimize growth-related variability. Selected isolates were also examined in 24-well plates for visual confirmation by inverted microscopy, as shown in **Supplementary Figure 1**.

### 2.5 DNA extraction and PCR gene detection

Genomic DNA was extracted from overnight cultures using the QIAamp DNA Mini Kit (Qiagen, Hilden, Germany) per the manufacturer’s instructions and quantified by NanoDrop 2000 spectrophotometry (Thermo Fisher Scientific, USA). DNA purity was confirmed by A260/A280 ratios of 1.8-2.0. PCR assays detected the intrinsic *bla*OXA-51-like gene (species confirmation) (J. F. et al., 2006), acquired carbapenemase genes (*blaOXA-23, blaOXA-24, blaOXA-58, blaNDM-1, and blaVIM, blaIMP*), β-lactamase genes (*blaPER-1*, AmpC, and *blaTEM*), and virulence genes (*bap, ompA, and csuE*). Primer sequence and thermocycling conditions followed validated published protocols mentioned in **Supplementary Table 2** and bands with amplicon size shown in **Supplementary Figures 2**, **3**. Reactions were performed in 25-μL volumes using Promega 2× GoTaq Master Mix (Promega, Madison, USA), with initial denaturation at 95 °C for 5 min, 30 amplification cycles (denaturation: 94 °C for 45 s; gene-specific annealing: 50 °C-60 °C for 1 min; extension: 72 °C for 1 min), and a final extension at 72 °C for 10 min.

Amplification products were resolved on 1.5% ethidium bromide-stained agarose gels alongside a 100-bp molecular weight ladder. Previously confirmed gene-positive isolates were used as positive controls wherever available, and molecular-grade water was included as a no-template negative control in every PCR run. PCR results were accepted only when the positive control showed the expected amplicon, the negative control showed no amplification, and the test isolate produced a clear band of the expected size. Weak, ambiguous, or non-specific bands were repeated from freshly prepared DNA.

One isolate yielded non-interpretable csuE PCR results despite repeat amplification and was excluded from csuE-specific analyses; therefore, csuE results were analyzed using a valid N of 199. Other PCR targets from this isolate amplified successfully, suggesting a target-specific technical issue rather than general DNA extraction failure. Representative amplicons are shown in **Supplementary Figures 2**, **3**.

### 2.6 Statistical analysis

All statistical analyses were performed using IBM SPSS Statistics version 25.0 (IBM Corp., Armonk, NY, USA). Data normality was assessed by the Shapiro–Wilk test; the non-normal distribution of MIC and resistance burden data necessitated non-parametric methods throughout. Group differences in MIC and resistance burden across biofilm categories were assessed by the Kruskal–Wallis test with Dunn–Bonferroni *post-hoc* pairwise comparisons; Spearman’s rank correlation coefficient (ρ) assessed monotonic associations. Categorical associations between gene presence and phenotypic resistance were evaluated using Pearson χ^2^ test or Fisher’s exact test where expected cell counts were <5.

Because environmental isolates may differ from clinical isolates in selection pressure and biofilm behavior, source-stratified analyses were performed by comparing clinical and environmental isolates separately. Primary analyses were also checked after excluding environmental isolates to assess whether the main genotype–phenotype and biofilm–resistance findings remained consistent.

Logistic regression was used to identify predictors of carbapenem resistance, and results were reported as odds ratios (ORs) with 95% confidence intervals (CIs). Genes showing complete separation or very low prevalence were excluded from regression modeling and analyzed descriptively using Fisher’s exact test. Very large ORs with wide confidence intervals were interpreted cautiously because they may reflect sparse data or complete-separation effects.

Genotype–phenotype concordance was assessed using Cohen’s κ coefficient, interpreted as >0.80 almost perfect, 0.61-0.80 substantial, 0.41-0.60 moderate, 0.21-0.40 fair, and ≤0.20 slight agreement. For diagnostic-performance analysis, complete 2×2 contingency tables were generated for blaOXA-23 and composite carbapenemase gene carriage using true positives, false positives, false negatives, and true negatives. Sensitivity, specificity, positive predictive value, and negative predictive value were calculated from these 2×2 tables. A two-sided p-value <0.05 was considered statistically significant.

## 3 Results

### 3.1 Isolate characteristics and source distribution

The 200 isolates originated from urine (18.0%), blood (15.5%), environmental swabs (14.0%), CSF (13.5%), sputum (11.5%), pus (10.0%), rectovaginal swabs (10.0%), and BALF (7.5%). Resistance classification revealed 54.0% MDR, 34.0% XDR, 1.0% PDR, and 11.0% non-MDR isolates; carbapenem resistance was present in 61.0%. The majority of isolates were strong biofilm formers (60.5%), followed by moderate (26.5%) and weak (13.0%) producers.

Because environmental isolates may differ from clinical isolates in selection pressure and biofilm behavior, source-stratified analysis was performed by comparing clinical isolates (n = 172) and environmental isolates (n = 28). The distribution of carbapenem resistance, MDR/XDR/PDR status, blaOXA-23 carriage, strong biofilm formation, resistance burden, and colistin MIC was compared between the two groups. The primary genotype–phenotype and biofilm–resistance findings remained consistent after considering isolate source. As only 28 isolates were environmental, source-stratified statistical analysis had limited power. Therefore, isolate source was considered during interpretation, and the pooling of clinical and environmental isolates has been acknowledged as a limitation.

Full baseline characteristics are summarized in **Table 1**.

**Table 1:** Baseline characteristics of *A. baumannii* isolates (N = 200).

Variable	Category	n (%)
Specimen type	BALF	15 (7.5)
	Blood	31 (15.5)
	CSF	27 (13.5)
	Environmental	28 (14.0)
	Pus	20 (10.0)
	Rectovaginal swab	20 (10.0)
	Sputum	23 (11.5)
	Urine	36 (18.0)
Biofilm formation	Weak	26 (13.0)
	Moderate	53 (26.5)
	Strong	121 (60.5)
Resistance category	Non-MDR	22 (11.0)
	MDR	108 (54.0)
	XDR	68 (34.0)
	PDR	2 (1.0)
Carbapenem resistance	Susceptible	78 (39.0)
	Resistant	122 (61.0)

n, number; BALF, broncho-alveolar lavage fluid; CSF, cerebrospinal fluid; MDR, multidrug-resistant; XDR, extensively drug-resistant; PDR, pan drug-resistant. Percentages calculated from total N = 200.

### 3.2 Antimicrobial susceptibility profile

Resistance rates were high across all major antibiotic classes (**Supplementary Table 1**). Fluoroquinolone resistance was near-universal (84.0% for ciprofloxacin and levofloxacin each). Carbapenem resistance was substantial and equivalent for both agents (meropenem and imipenem, 60.5% each). Aminoglycoside resistance was high (amikacin 67.5%, gentamicin 58.5%), as was cephalosporin resistance (74.5%-75.5%).

**Figure 1:**
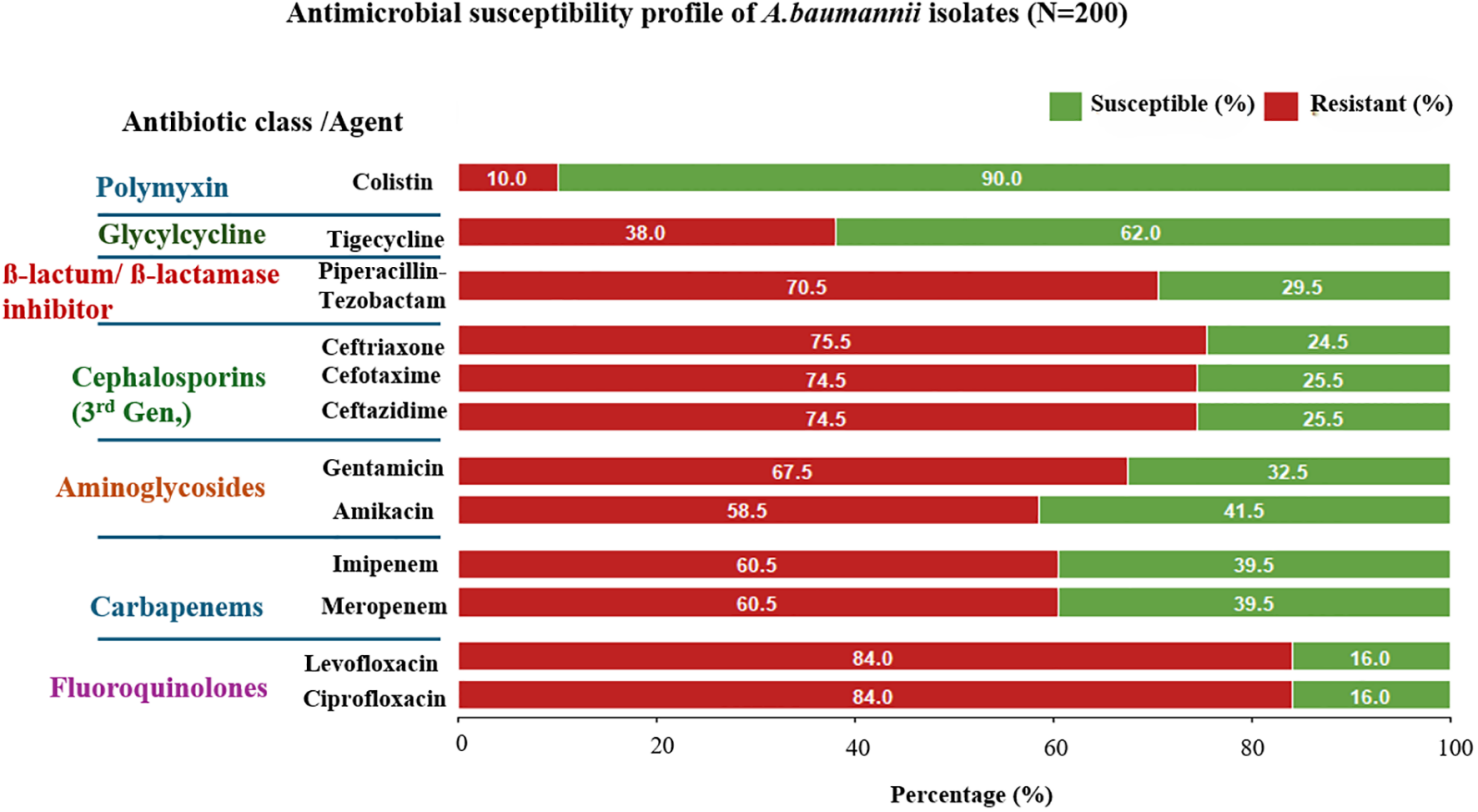
Antimicrobial susceptibility profile of *A. baumannii* isolates (N = 200).

Colistin retained activity against most isolates, with 90.0% of isolates categorized as susceptible according to EUCAST criteria. Using the EUCAST colistin resistance breakpoint of >2 mg/L, 20/200 isolates were categorized as colistin-resistant. Tigecycline showed susceptibility in 62.0% of isolates (**Figure 1**).

### 3.3 Gene distribution

**Figure 2:**
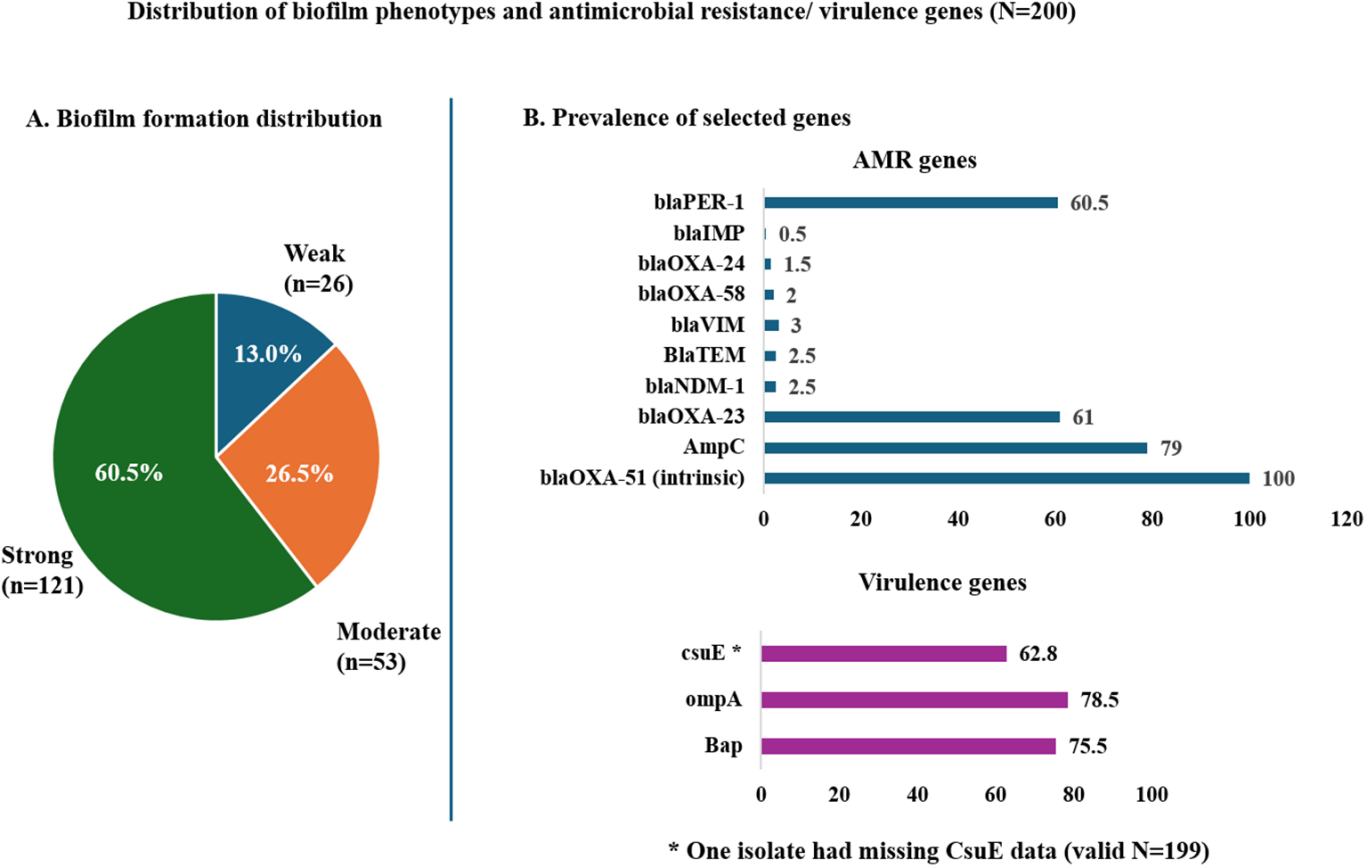
**(A)** Biofilm forming capacity of the isolates. **(B)** Prevalence of antimicrobial resistance genes (ARGs) and virulence genes among *A. baumannii* isolates (N = 200).

All 200 isolates carried the intrinsic *bla*OXA-51 gene, confirming species identity. Among acquired carbapenemase genes, *blaOXA-23* predominated at 61.0%; other carbapenemase genes were detected at low frequencies (≤3%) (≤3.0% each). AmpC was widespread (79.0%), and *blaPER-1* was detected in 60.5% of isolates. Virulence gene prevalence was high: *ompA* 78.5%, *bap* 75.5%, and *csuE* 62.8%. One isolate had non-interpretable csuE PCR results and was excluded from csuE-specific analysis; therefore, csuE prevalence was calculated using a valid denominator of 199. The distribution of antimicrobial resistance and virulence genes is summarized in **Supplementary Table 3** and illustrated in **Figure 2**.

### 3.4 Biofilm formation versus antimicrobial resistance burden

**Figure 3:**
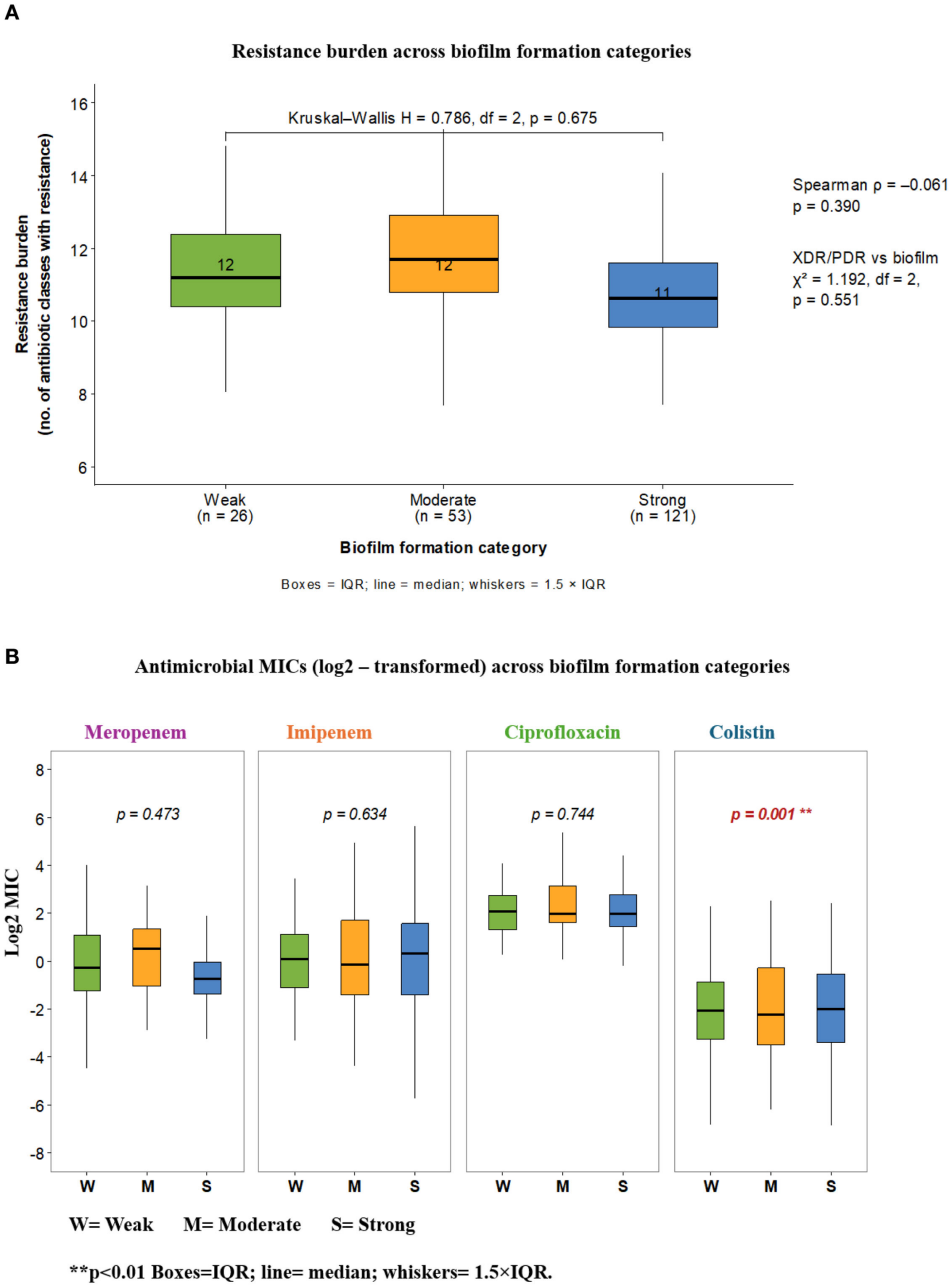
**(A)** Distribution of antimicrobial resistance burden across biofilm formation categories in *A. baumannii* isolates. Box-and-whisker plot showing resistance burden (number of antibiotic classes with resistance) among weak, moderate, and strong biofilm-forming *A. baumannii* isolates. Median values and interquartile ranges are indicated. No statistically significant difference was observed between biofilm categories (Kruskal–Wallis test, p > 0.05). **(B)** Comparative antimicrobial MIC distributions according to biofilm formation capacity. Boxplots illustrating log_2_-transformed MIC distributions of meropenem, imipenem, ciprofloxacin, and colistin among weak, moderate, and strong biofilm-forming *A. baumannii* isolates. Strong biofilm producers demonstrated significantly elevated colistin MIC values, whereas no significant differences were observed for meropenem, imipenem, or ciprofloxacin.

Resistance burden did not differ significantly across weak, moderate, and strong biofilm-forming isolates, and no significant association was observed between biofilm formation capacity and cumulative antimicrobial resistance (**Figure 3A**). Similarly, XDR/PDR status showed no significant relationship with biofilm phenotype. Resistance burden (number of antibiotic classes with resistance) did not differ significantly across biofilm categories: median 12 (IQR 10.75-13) for weak, 12 (IQR 9-13) for moderate, and 11 (IQR 8-13) for strong producers. Kruskal–Wallis analysis confirmed no significant group difference (H = 0.786, df = 2, p = 0.675), and Spearman correlation showed no monotonic association (ρ = −0.061, p = 0.390) (**Table 2**). Chi-square analysis of XDR/PDR status versus biofilm category was likewise non-significant (χ^2^ = 1.192, df = 2, p = 0.551).

**Table 2:** Association of virulence-associated genes with biofilm formation phenotypes in *A. baumannii*.

Gene	Status	Weak n (%)	Moderate n (%)	Strong n (%)	p-value
*bap*	Absent (n=49)	20 (40.8)	27 (55.1)	2 (4.1)	**<0.001**
	Present (n=151)	6 (4.0)	26 (17.2)	119 (78.8)	
*ompA*	Absent (n=43)	17 (39.5)	24 (55.8)	2 (4.7)	**<0.001**
	Present (n=157)	9 (5.7)	29 (18.5)	119 (75.8)	
*csuE*	Absent (n=74)	17 (23.0)	29 (39.2)	28 (37.8)	**<0.001**
	Present (n=125)	9 (7.2)	23 (18.4)	93 (74.4)	
*blaPER-1*	Absent (n=79)	20 (25.3)	29 (36.7)	30 (38.0)	**<0.001**
	Present (n=121)	6 (5.0)	24 (19.8)	91 (75.2)	

Pearson χ^2^ test used for comparison across biofilm categories. One isolate had missing csuE data (valid N = 199). Bold values indicate statistically significant results (p < 0.05).

MIC analysis showed a significant association only for colistin, with strong biofilm producers showing higher colistin MIC values compared with weak producers (p = 0.001). In contrast, no significant MIC differences were observed for meropenem, imipenem, or ciprofloxacin across biofilm categories. Using the EUCAST colistin resistance breakpoint of >2 mg/L, 20/200 isolates were categorized as colistin-resistant. Therefore, the observed colistin association reflects both categorical resistance in a subset of isolates and an upward MIC shift among strong biofilm producers. Growth-curve analysis was not performed in parallel with the biofilm assay; therefore, growth-rate effects on OD570-based biofilm biomass or MIC comparisons cannot be fully excluded. However, inoculum density, medium composition, incubation time, and assay conditions were standardized across all isolates.

Comparative MIC distributions across biofilm categories are summarized in **Table 3** and illustrated in **Figure 3**.

**Table 3:** Comparison of antimicrobial MIC distributions across biofilm formation categories.

Antibiotic	Weak (IQR)	Moderate (IQR)	Strong (IQR)	p-value
Meropenem	3.5 (−1.0–5.0)	4.0 (−0.5–5.0)	3.0 (−1.0–5.0)	0.473
Imipenem	3.5 (−1.0–5.0)	4.0 (−1.0–5.0)	4.0 (−1.0–5.0)	0.634
Ciprofloxacin	4.0 (4.0–5.0)	4.0 (3.5–5.0)	4.0 (4.0–5.0)	0.744
Colistin	−3.0 (−3.0 to −2.0)	−3.0 (−4.06 to −2.0)	−2.0 (−3.0 to −2.0)	**0.001** **

MIC values are expressed as median log_2_ MIC with interquartile range (IQR). Statistical comparisons were performed using the Kruskal–Wallis test. Only colistin demonstrated a significant association with biofilm formation capacity (p < 0.01). Bold values indicate statistically significant results (p < 0.05).

### 3.5 Carbapenemase gene–phenotype associations

**Figure 4:**
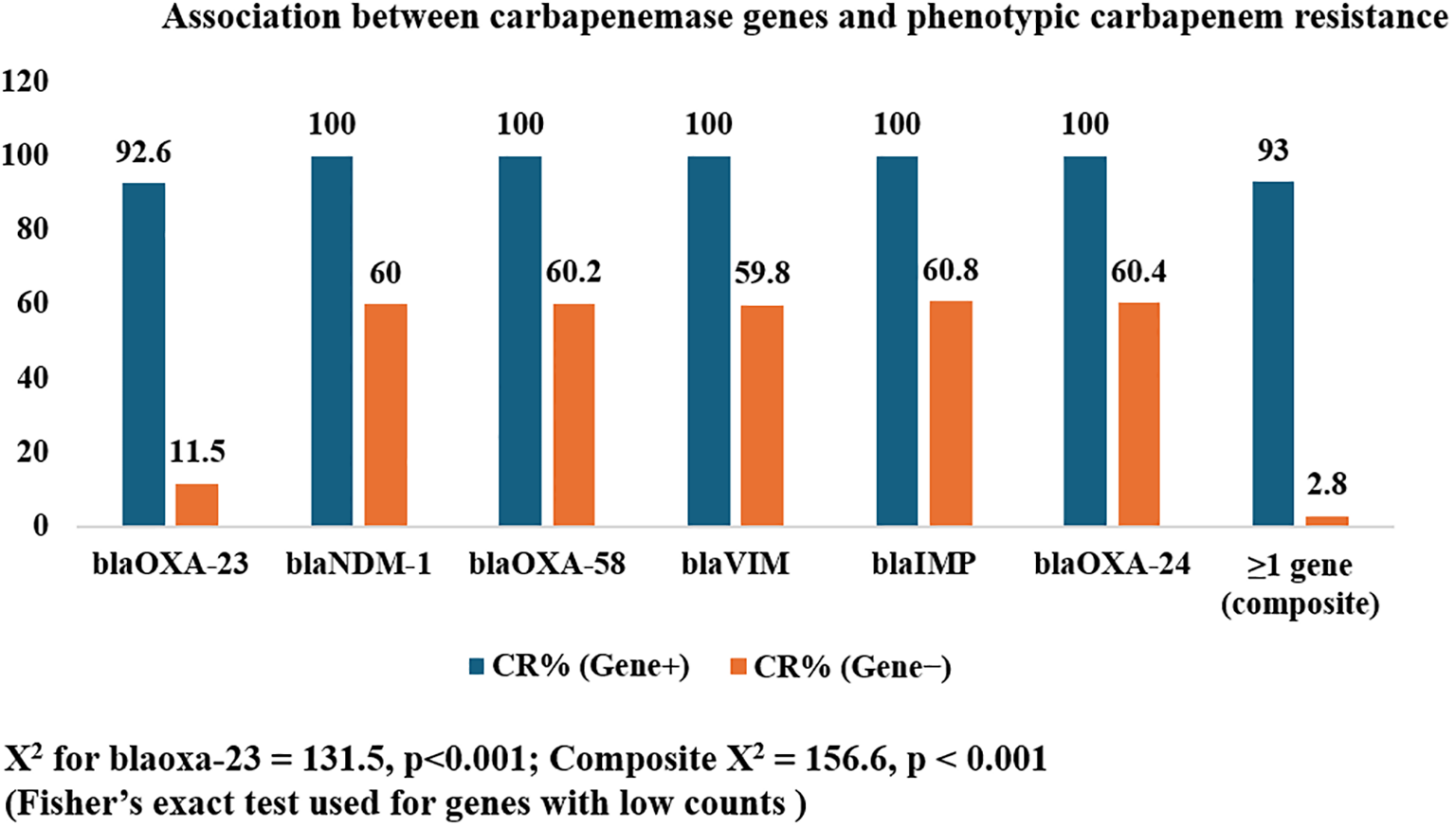
Relationship between carbapenemase gene carriage and phenotypic carbapenem resistance. Distribution of phenotypic carbapenem resistance among isolates carrying individual and composite carbapenemase genes. blaOXA-23 and composite carbapenemase gene carriage demonstrated strong associations with carbapenem resistance, whereas minor carbapenemase genes were detected at low frequencies.

The *blaOXA-23* gene was strongly and significantly associated with carbapenem resistance: 92.6% of gene-positive isolates were resistant versus 11.5% of gene-negative isolates (χ^2^ = 131.5, p < 0.001; OR = 96.26, 95% CI 36.45-254.24) (**Figure 4**). A small subset of blaOXA-23-positive isolates remained carbapenem-susceptible, indicating genotype–phenotype discordance. These discordant findings may reflect absence of upstream ISAba1 activation, low gene expression, or other regulatory mechanisms; however, ISAba1 mapping and expression analysis were not performed.

Composite carbapenemase gene carriage, defined as the presence of at least one carbapenemase gene, showed a strong association with carbapenem resistance, with resistance rates of 93.0% in gene-positive isolates and 2.8% in gene-negative isolates (χ^2^ = 156.6, p < 0.001; OR = 460.0, 95% CI 96.6–2190.2). Because the composite OR had a very wide confidence interval, this estimate was interpreted cautiously and considered supportive rather than definitive. Carbapenemase gene burden showed a strong positive correlation with carbapenem MIC escalation, particularly for meropenem (Spearman ρ = 0.816, p < 0.001).

### 3.6 Genotype–phenotype concordance

*blaOXA-23* demonstrated almost-perfect concordance with phenotypic carbapenem resistance (κ = 0.81; sensitivity 92.6%, specificity 88.5%, positive predictive value [PPV] 92.6%, negative predictive value [NPV] 88.5% (**Table 4**).

**Table 4:** Association of carbapenemase genes with phenotypic carbapenem resistance in *A. baumannii*.

Gene / group	CR% (Gene+)	CR% (Gene−)	Test	Statistic	P-value
*bla*OXA-23	92.6	11.5	χ²	131.5	<0.001
*bla*NDM-1	100	60.0	Fisher's exact	—	0.159
*bla*OXA-58	100	60.2	Fisher's exact	—	0.158
*bla*VIM	100	59.8	Fisher's exact	—	0.083
*bla*IMP	100	60.8	Fisher's exact	—	1.000
*bla*OXA-24	100	60.4	Fisher's exact	—	0.283
≥1 gene (composite)	93.0	2.8	χ²	156.6	<0.001

CR, carbapenem resistant. Fisher's exact test applied where expected cell count < 5. Complete separation precluded OR estimation for minor genes. χ², Pearson chi-square; CI, confidence interval.

To avoid ambiguity in diagnostic-performance calculations, complete 2×2 contingency data were generated for blaOXA-23 and composite carbapenemase gene carriage (**Table 5**). For blaOXA-23, the equality between sensitivity and PPV, and between specificity and NPV, resulted from identical marginal totals for blaOXA-23 carriage and carbapenem resistance in this dataset.

**Table 5:** Diagnostic performance and genotype-phenotype concordance of carbapenemase genes for prediction of carbapenem resistance in *A. baumannii*.

Gene/group	TP	FP	FN	TN	Sensitivity (%)	Specificity (%)	PPV (%)	NPV (%)	Cohen’s κ
blaOXA-23	113	9	9	69	92.6	88.5	92.6	88.5	0.81
blaNDM-1	5	0	117	78	4.1	100.0	100.0	40.0	NE
blaOXA-58	4	0	118	78	3.3	100.0	100.0	39.8	NE
blaVIM	6	0	116	78	4.9	100.0	100.0	40.2	NE
blaIMP	1	0	121	78	0.8	100.0	100.0	39.2	NE
blaOXA-24	3	0	119	78	2.5	100.0	100.0	39.6	NE
≥1 carbapenemase gene	120	9	2	69	98.4	88.5	93.0	97.2	0.88

*TP, true positive; FP, false positive; FN, false negative; TN, true negative; PPV, positive predictive value; NPV, negative predictive value; NE, not estimable due to sparse data/complete separation. Phenotypic carbapenem resistance was used as the reference standard. For blaOXA-23, identical marginal totals for blaOXA-23 carriage and phenotypic carbapenem resistance explain why sensitivity equals PPV and specificity equals NPV.

A small number of blaOXA-23-positive isolates were phenotypically susceptible to carbapenems, indicating genotype–phenotype discordance. Because ISAba1 mapping, gene-expression analysis, and WGS were not performed, the mechanism of this discordance could not be confirmed. Possible explanations include absence of upstream ISAba1 promoter activation, low-level gene expression, or other regulatory effects.

Minor carbapenemase genes showed perfect specificity but near-zero sensitivity; Cohen’s κ was not estimable due to sparse data resulting in complete separation (**Table 5**). AmpC demonstrated moderate concordance with extended-spectrum β-lactam resistance (κ = 0.575, p < 0.001), while *blaTEM* showed negligible concordance (κ = 0.006), consistent with its variable expression in *A. baumannii*. The universal *bla*OXA-51 gene showed wide variation in carbapenem MIC, confirming that intrinsic oxacillinase activity alone is insufficient for clinical resistance without upstream ISAba1 promoter activation.

### 3.7 Virulence–resistance–biofilm triangle

All three virulence genes were strongly and independently associated with strong biofilm formation: *bap* (78.8% vs. 4.1% strong-producer prevalence in gene-present vs. gene-absent; χ^2^ = 92.81, p < 0.001), *ompA* (75.8% vs. 4.7%; χ^2^ = 75.67, p < 0.001), and *csuE* (74.4% vs. 37.8%; χ^2^ = 26.76, p < 0.001). Presence of *blaPER-1* was similarly associated with strong biofilm formation (75.2% vs. 38.0%; χ^2^ = 31.32, p < 0.001) (**Table 2**).

Although virulence-associated genes were strongly linked to biofilm formation, they were not associated with increased antimicrobial resistance burden. Mann–Whitney U analysis showed no significant difference in resistance burden according to bap, ompA, csuE, or blaPER-1 carriage (all p > 0.05) (**Table 6**). These findings support functional separation between the virulence–biofilm axis and the antimicrobial-resistance axis in this isolate collection. However, because MLST/WGS was not performed, possible clonal effects cannot be excluded.

**Table 6:** Relationship between virulence gene carriage and antimicrobial resistance burden.

Virulence gene	Status	N	Median burden (IQR)	p-value (MWU)
*bap*	Absent	49	12 (9-13)	0.383
	Present	151	12 (8-13)	
*ompA*	Absent	43	12 (9-13)	0.627
	Present	157	12 (8-13)	
*csuE*	Absent	74	11 (8-13)	0.519
	Present	125	12 (9-13)	
*blaPER-1*	Absent	79	11 (8-13)	0.461
	Present	121	12 (9-13)	

MWU, Mann–Whitney U test; resistance burden defined as the number of antibiotic classes showing resistance.

No significant association was observed between virulence gene carriage and antimicrobial resistance burden for any gene analyzed (all Mann–Whitney U, p > 0.05).

## 4 Discussion

This study presents one of the most comprehensively genotyped *A. baumannii* datasets from Haryana, Delhi-NCR part of northern India to date, and highlights a clinically important finding: carbapenem resistance and biofilm formation largely behave as independent adaptive traits. While blaOXA-23 strongly associated with carbapenem resistance, virulence-associated genes such as bap, ompA, and csuE were primarily linked to enhanced biofilm formation and reduced colistin susceptibility rather than broader antimicrobial resistance.

### 4.1 Regional AMR burden in global context

The high burden of carbapenem resistance and XDR phenotypes observed in this study is similar to surveillance data from the ATLAS surveillance network, which identified nearly 87% carbapenem-resistant *A. baumannii* across South and Southeast Asia (Murray et al., 2022). These resistance levels are higher than those observed in Europe and North America, where rates of carbapenem resistance remain far lower (Lee et al., 2023). A closely related study from Kathmandu Model Hospital, Nepal, showed 81.45% carbapenem resistance in the *Acinetobacter calcoaceticus–baumannii* complex, mostly attributed to blaOXA-23-like genes (Bhandari et al., 2024; Gurung et al., 2024). Another tertiary-care study from Telangana, India, similarly reported extremely high MDR (91.6%) and XDR (88.1%) rates among *Acinetobacter* isolates (Sultana et al., 2025). The high fluoroquinolone resistance rate observed in the present *A. baumannii* collection, with 84.0% resistance to both ciprofloxacin and levofloxacin, likely reflects antibiotic selection pressure and circulation of resistant hospital-adapted lineages. However, because MLST or WGS was not performed, the contribution of specific clonal lineages could not be determined (Saksena et al., 2018).

### 4.2 blaOXA-23: the molecular engine of carbapenem resistance

The strong concordance between blaOXA-23 carriage and phenotypic carbapenem resistance supports its role as a major predictor of CRAB in this northern Indian isolate collection. This concordance exceeds that reported in many prior studies and aligns with evidence from global surveillance confirming the dominant role of *blaOXA-23* in CRAB epidemiology (a gene carried on conjugative plasmids in the globally dominant CC2 clonal lineage and mobilized by ISAba1 insertion sequences that provide outward-facing promoter activity) (Boutsoukas and Doi, 2025; Shahari et al., 2025). However, because MLST, WGS, ISAba1 mapping, and blaOXA-23 expression analysis were not performed, the observed association cannot be interpreted as definitive evidence of a gene-level causal relationship independent of clonal background.

This conclusion is backed by comparative international evidence. Ying et al. characterized CRAB isolates in China and found that bla_OXA-23_ genes were present in all CRAB isolates (Chen et al., 2017). Wang et al. (2024) similarly identified *blaOXA-23* in 74.5% of Chinese CRAB isolates, reporting significant variation among biofilm formation categories (p < 0.05) (Wang et al., 2024). This finding contrasts with the present study, where biofilm formation was not associated with cumulative antimicrobial resistance burden. The discrepancy may reflect differences in isolate source, sampling structure, local antimicrobial exposure, biofilm assay conditions, or underlying clonal composition. However, because clonal typing was not performed in the present study, clonal lineage differences remain only a possible explanation and cannot be confirmed. Minor carbapenemase genes were detected only sporadically, suggesting a limited contribution to population-level resistance in this setting.

From the evolutionary and structural points of view, OXA-23 and OXA-58 belong to the class D carbapenem-hydrolyzing β-lactamase (CHDL) family and exhibit ~60%-70% amino acid identity and 75%–85% similarity (Kaitany et al., 2013), indicative of conservation of the catalytic β-lactamase scaffold despite functionally relevant divergence (Poirel et al., 2010). Identity is the exact match of amino acids in a sequence. Similarity includes conservative substitutions, where amino acids are replaced by others with similar physicochemical properties and partial conservation of function. The largest family of CHDL is the OXA-23-like, with more than 150 different variants described (Evans and Amyes, 2014), which suggests a great evolutionary fitness and a wide spread in international high-risk clones. In contrast, the OXA-58-like family has fewer and more geographically restricted variants. Despite their evolutionary relationship, these enzymes exhibit differences in regulatory architecture and expression dynamics, which influence carbapenem-hydrolyzing efficiency and epidemiological prevalence (NT et al., 2014). Taken together, these data indicate that blaOXA-23 is the main carbapenem resistance determinant in our isolates, while blaOXA-58 could be a secondary and context-dependent determinant.

From a diagnostic standpoint, these findings powerfully support the integration of *blaOXA-23* PCR ideally into frontline resistance screening. Rapid molecular platforms including commercial OXA-specific PCR cartridges can deliver results within 1–2 h of isolate recovery. Nevertheless, blaOXA-23 detection should complement rather than replace phenotypic AST, because a small number of blaOXA-23-positive isolates remained carbapenem-susceptible and mechanisms such as ISAba1-mediated promoter activation or gene-expression variation were not assessed in this study.

### 4.3 Biofilm–resistance decoupling: evidence and mechanism

The absence of a meaningful relationship between biofilm formation and resistance burden challenges the commonly assumed link between multidrug resistance and enhanced biofilm production in *A. baumannii*. This null result is not a statistical artefact: the collection of 200 isolates is adequately powered, and convergent results across both continuous burden analysis and categorical MDR/XDR classification confirm robustness. In the present study, resistance burden did not differ significantly across weak, moderate, and strong biofilm-forming isolates, and XDR/PDR status was not significantly associated with biofilm category.

This finding is further corroborated by recent independent evidence. Biofilm formation was present in 99% of isolates, but biofilm formation was not directly correlated with resistance phenotype—247 *A. baumannii* strains isolated from Chennai, India (2025) (Kannan et al., 2025). Likewise, Jyoti et al. in their collection reported that 91% CRAB has *bla*_OXA23-like_ genes but no linkage between biofilm forming capacity and resistance spectrum (Choudhary and Shariff, 2025), considering these globally reported observations which indicate that these two aspects—biofilm formation and antibiotic resistance—are not dependent on each other and are adaptive traits of *A. baumannii*.

However, growth kinetics were not assessed in parallel with the biofilm assay in the present study. Therefore, although inoculum density and assay conditions were standardized, the possibility that differences in bacterial growth rate influenced OD570-based biofilm biomass or MIC comparisons cannot be fully excluded.

The only notable exception was the reduced colistin susceptibility observed among strong biofilm producers. This observation is mechanistically explainable: colistin (polymyxin E) exerts its effect by electrostatic exchange of divalent cations (Ca^2+^, Mg^2+^) from the lipid A part of lipopolysaccharide (LPS), leading to destabilization of outer membrane. In particular, the anionic EPS matrix of *A. baumannii* biofilms—which contains poly-β-(1,6)-N-acetylglucosamine, alginate-like polysaccharides, and extracellular DNA—sequesters these cations and physically prevents colistin from reaching its site of action at the bacterial surface. This may represent a biofilm-associated phenotypic tolerance mechanism rather than confirmed genetic colistin resistance, as pmrAB mutations, lpxA/C/D alterations, and other molecular mechanisms of colistin resistance were not investigated in this study (Beceiro et al., 2011). The practical implication is direct: routine colistin MIC testing performed on planktonic bacteria will systematically underestimate the effective concentration required to eradicate biofilm-associated *A. baumannii* infections. Therefore, a colistin MIC within the susceptible range may not fully exclude reduced treatment efficacy in biofilm-associated infections, particularly in device-associated infections or VAP.

### 4.4 The virulence–biofilm axis: robust but resistance-independent

The strong association of bap, ompA, and csuE with biofilm formation supports their established role in adhesion and biofilm maturation. The lack of relationship between virulence-associated genes and resistance burden further supports the concept that virulence and antimicrobial resistance may evolve independently under different hospital selection pressures. However, because MLST or WGS was not performed, possible clonal effects on the distribution of virulence genes and biofilm phenotypes cannot be excluded.

### 4.5 Implications for diagnostics, therapy, and infection control

Three clinically actionable recommendations emerge from this study. First, blaOXA-23 PCR may be useful as a rapid screening tool in high-CRAB settings; however, it should complement rather than replace phenotypic AST because genotype–phenotype discordance was observed in a small subset of isolates. Second, phenotypic biofilm quantification may be considered as a supplementary research or risk-stratification tool in device-associated *A. baumannii* infections, particularly where colistin therapy is contemplated, because strong biofilm formers showed elevated colistin MICs. Third, the high prevalence of virulence-gene-driven biofilm capacity underscores the need for anti-biofilm adjunct strategies—including biofilm-disrupting adjuncts, improved device coatings, and newer combination therapies for multidrug-resistant *A. baumannii* (Hibstu et al., 2022), surface-active coatings on indwelling devices, and combination regimens pairing cefiderocol or sulbactam–durlobactam with biofilm-disrupting agents (Karruli et al., 2023). These diagnostic and therapeutic implications should be interpreted cautiously because MLST/WGS, ISAba1 mapping, gene-expression analysis, and clinical outcome correlation were not performed.

### 4.6 Limitations

Limitations of this study are that, first, the retrospective nature of the study using archived clinical isolates did not allow for longitudinal monitoring of resistance trends or correlation with patient outcomes, and second, the isolates were obtained from a single geographical area in Northern India, and this may restrict the wider epidemiological significance of the findings. In addition, clinical and environmental isolates were analyzed together; therefore, differences in selection pressure between patient-derived and environmental isolates may have influenced some associations. Third, advanced molecular typing approaches such as multilocus sequence typing (MLST) and whole-genome sequencing (WGS) were not undertaken. As a result, clonal relationships, international lineage assignments, plasmid-mediated transmission, resistance islands, insertion sequence mapping (particularly ISAba1 upstream of blaOXA genes), and other mobile genetic elements driving resistance dissemination could not be fully delineated. Species confirmation was based on conventional microbiological characterization and blaOXA-51-like PCR; MALDI-TOF MS or 16S rRNA sequencing was not performed. ISAba1 mapping upstream of blaOXA genes and gene-expression analysis were also not performed; therefore, mechanisms underlying blaOXA-23-positive but carbapenem-susceptible discordant isolates could not be determined. Growth kinetics were not assessed in parallel with the biofilm assay; therefore, growth-rate effects on OD570-based biofilm biomass or MIC comparisons across biofilm categories cannot be fully excluded. Finally, clinical outcome data were unavailable, preventing correlation of genotype, biofilm phenotype, and MIC findings with patient-level treatment outcomes.

### 4.7 Future perspectives

Future multicenter prospective studies integrating whole-genome sequencing (WGS), transcriptomic profiling, and detailed clinical outcome data are needed to better understand the molecular evolution, transmission dynamics, and therapeutic behavior of CRAB isolates. Future work should also include ISAba1 mapping upstream of blaOXA genes, blaOXA-23 expression analysis, and source-stratified comparisons of clinical and environmental isolates to clarify genotype–phenotype discordance and possible clonal confounding. Growth-kinetic assessment and CFU-based validation should be incorporated alongside crystal violet biofilm assays to exclude growth-rate effects on biofilm and MIC comparisons. These approaches may also help in the identification of clinically relevant resistance-virulence markers and enable the development of more targeted diagnostic and precision-treatment strategies for biofilm-associated carbapenem-resistant *A. baumannii* infections.

## 5 Conclusions

This study demonstrates with statistical precision that biofilm formation and carbapenem resistance diverge as functionally independent adaptive strategies in *A. baumannii*. The *blaOXA-23* carbapenemase is the indisputable molecular engine of carbapenem resistance (κ = 0.81, OR 96.26), validating its use as a rapid diagnostic marker in high-CRAB settings. Biofilm formation, mediated by Bap, OmpA, and CsuE, establishes a distinct persistence mechanism that, while conferring clinically significant tolerance to colistin through EPS-mediated cation sequestration, does not expand the spectrum of conventional antimicrobial resistance. Understanding this decoupling requires a paradigm shift in our interpretation of *A. baumannii* clinical isolates: resistance genotyping and biofilm phenotyping give complementary but not redundant information, uniquely required to optimize stratification of infection severity and thereby therapeutic approach. Future WGS-integrated, prospective studies which connect clonal epidemiology with clinical outcomes are key to adapting these insights for precision infection management.

OR

This study suggests that biofilm formation and carbapenem resistance may represent partially independent adaptive traits in *A. baumannii*. blaOXA-23 showed strong concordance with phenotypic carbapenem resistance and may serve as a useful rapid molecular marker in high-CRAB settings. However, because MLST, WGS, ISAba1 mapping, and gene-expression analyses were not performed, clonal confounding and variable gene expression cannot be excluded. Biofilm formation, associated with bap, ompA, and csuE carriage, appeared to contribute more strongly to persistence and colistin MIC elevation than to broad multidrug resistance. These findings support the complementary use of resistance genotyping and biofilm phenotyping for improved risk stratification of *A. baumannii* isolates. Future multicenter studies integrating WGS, transcriptomics, growth-kinetic assessment, and clinical outcome data are required to validate these observations and determine their therapeutic relevance.

## Data Availability

The original contributions presented in the study are included in the article/**Supplementary Material**. Further inquiries can be directed to the corresponding author.

## References

[B1] Antimicrobial Resistance Collaborators (2022). Global burden of bacterial antimicrobial resistance in 2019: a systematic analysis. Lancet 399 (10325), 629–655. doi: 10.1016/S0140-6736(21)02724-0 35065702 PMC8841637

[B2] Beceiro A. . (2011). Phosphoethanolamine modification of lipid A in colistin-resistant variants of Acinetobacter baumannii mediated by the pmrAB two-component regulatory system. Antimicrob. Agents Chemother. 55, 3370–3379. doi: 10.1128/AAC.00079-11 21576434 PMC3122444

[B3] Bhandari S. Upreti M. K. Angbuhang K. B. Shrestha B. Thapa Shrestha U. (2024). Increased biofilm-associated carbapenem-resistant Acinetobacter calcoaceticus-baumannii complex infections among hospitalised patients in Kathmandu Model Hospital, Nepal. J. Global Antimicrob. Resist. 39, 1–2. doi: 10.1016/j.jgar.2024.07.012 39097145

[B4] Boutsoukas A. Doi Y. (2025). The global epidemiology of carbapenem-resistant Acinetobacter baumannii. JAC-antimicrobial Resist. 7, dlaf134. doi: 10.1093/jacamr/dlaf134 40735512 PMC12305305

[B5] Brossard K. A. Campagnari A. A. (2012). The Acinetobacter baumannii biofilm-associated protein plays a role in adherence to human epithelial cells. Infect. Immun. 80, 228–233. doi: 10.1128/IAI.05913-11 22083703 PMC3255684

[B6] Burcu I. Yohei D. B. R. A. P. D. L. (2018). New treatment options against carbapenem-resistant Acinetobacter baumannii infections. Antimicrob. Agents Chemother. 63, 10.1128/aac.01110–18. doi: 10.1128/aac.01110-18 30323035 PMC6325237

[B7] Chen Y. Gao J. Zhang H. Ying C. (2017). Spread of the bla(OXA-23)-containing Tn2008 in carbapenem-resistant Acinetobacter baumannii isolates grouped in CC92 from China. Front. Microbiol. 8, 163. doi: 10.3389/fmicb.2017.00163 28220115 PMC5292404

[B8] Choudhary J. Shariff M. (2025). Characterization of carbapenem-resistant biofilm forming Acinetobacter baumannii isolates from clinical and surveillance samples. Sci. Rep. 15, 33892. doi: 10.1038/s41598-025-09530-w 41028035 PMC12484597

[B9] CLSI C. L. S. I. (2015). Methods for dilution antimicrobial susceptibility tests for bacteria that grow aerobically; approved standard — ninth edition. CLSI document M07-A9. Clin. Lab. Standars Inst. 32, 18.

[B10] European Committee on Antimicrobial Susceptibility Testing . (2026). European Committee on Antimicrobial Susceptibility Testing Breakpoint tables for interpretation of MICs and zone diameters.

[B11] Evans B. A. Amyes S. G. B. (2014). OXA β-lactamases. Clin. Microbiol. Rev. 27, 241–263. doi: 10.1128/CMR.00117-13 24696435 PMC3993105

[B12] Gedefie A. Demsis W. Ashagrie M. Kassa Y. Tesfaye M. Tilahun M. . (2021). Acinetobacter baumannii biofilm formation and its role in disease pathogenesis: a review. Infect. Drug Resist. 14, 3711–3719. doi: 10.2147/IDR.S332051 34531666 PMC8439624

[B13] Gurung A. Napit R. Shrestha B. Lekhak B. (2024). Carbapenem resistance in Acinetobacter calcoaceticus-baumannii complex isolates from Kathmandu Model Hospital, Nepal, is attributed to the presence of blaOXA-23-like and blaNDM-1 genes. BioMed. Res. Int. 2024, 8842625. doi: 10.1155/2024/8842625 39161641 PMC11333142

[B14] Hibstu Z. Belew H. Akelew Y. Mengist H. M. (2022). Phage therapy: a different approach to fight bacterial infections. Biologics 16, 173–186. doi: 10.2147/BTT.S381237 36225325 PMC9550173

[B15] J. F. T. Neil W. Judith G. Susannah Y. E. K. M. T. L. P. (2006). Identification of Acinetobacter baumannii by detection of the blaOXA-51-like carbapenemase gene intrinsic to this species. J. Clin. Microbiol. 44, 2974–2976. doi: 10.1128/jcm.01021-06 16891520 PMC1594603

[B16] Kaitany K. C. J. Klinger N. V. June C. M. Ramey M. E. Bonomo R. A. Powers R. A. . (2013). Structures of the class D carbapenemases OXA-23 and OXA-146: mechanistic basis of activity against carbapenems, extended-spectrum cephalosporins, and aztreonam. Antimicrob. Agents Chemother. 57, 4848–4855. doi: 10.1128/AAC.00762-13 23877677 PMC3811470

[B17] Kannan K. P. Smiline Girija A. S. Priyadharsini V. J. (2025). Deciphering the twinning act of antimicrobial resistance and biofilm-associated virulence in multidrug-resistant Acinetobacterbaumannii. Biochem. Biophys. Res. Commun. 780, 152487. doi: 10.1016/j.bbrc.2025.152487 40818286

[B18] Karruli A. Migliaccio A. Pournaras S. Durante-Mangoni E. Zarrilli R. (2023). Cefiderocol and sulbactam-durlobactam against carbapenem-resistant Acinetobacter baumannii. Antibiot (Basel Switzerland) 12 (12), 1729. doi: 10.3390/antibiotics12121729 38136764 PMC10740486

[B19] Kyriakidis I. Vasileiou E. Pana Z. D. Tragiannidis A. (2021). Acinetobacter baumannii antibiotic resistance mechanisms. Pathogens 10, 1–31. doi: 10.3390/pathogens10030373 33808905 PMC8003822

[B20] Lee Y.-L. Ko W.-C. Hsueh P.-R. (2023). Geographic patterns of Acinetobacter baumannii and carbapenem resistance in the Asia-Pacific region: results from the Antimicrobial Testing Leadership and Surveillance (ATLAS) program, 2012-2019. Int. J. Infect. Dis. IJID Off. Publ Int. Soc Infect. Dis. 127, 48–55. doi: 10.1016/j.ijid.2022.12.010 36516915

[B21] Magiorakos A. P. Srinivasan A. Carey R. B. Carmeli Y. Falagas M. E. Giske C. G. . (2012). Multidrug-resistant, extensively drug-resistant and pandrug-resistant bacteria: an international expert proposal for interim standard definitions for acquired resistance. Clin. Microbiol. Infect. 18, 268–281. doi: 10.1111/j.1469-0691.2011.03570.x 21793988

[B22] Michaelis C. Grohmann E. (2023). Horizontal gene transfer of antibiotic resistance genes in biofilms. Antibiot (Basel Switzerland) 12 (2), 328. doi: 10.3390/antibiotics12020328 36830238 PMC9952180

[B23] Mohapatra S. S. Dwibedy S. K. Padhy I. (2021). Polymyxins, the last-resort antibiotics: mode of action, resistance emergence, and potential solutions. J. Biosci. 46, 93. doi: 10.1007/s12038-021-00209-8 34475315 PMC8387214

[B24] Murray C. J. L. Ikuta K. S. Sharara F. Swetschinski L. Aguilar G. R. Gray A. . (2022). Global burden of bacterial antimicrobial resistance in 2019: a systematic analysis. Lancet 399, 629–655. doi: 10.1016/S0140-6736(21)02724-0 35065702 PMC8841637

[B25] Najafabadi M. K. Soltani R. (2024). Carbapenem-resistant Acinetobacter baumannii and ventilator-associated pneumonia; epidemiology, risk factors, and current therapeutic approaches. J. Res. Pharm. Pract. 13, 33–40. doi: 10.4103/jrpp.jrpp_50_24 39830948 PMC11737613

[B26] NT Lamoureaux T. L. Toth M. Stewart N. K. Frase H. Vakulenko S. B. (2014). Class D β-lactamases: are they all carbapenemases? Antimicrob. Agents Chemother. 58, 2119–2125. doi: 10.1128/AAC.02522-13 24468778 PMC4023754

[B27] Poirel L. Naas T. Nordmann P. (2010). Diversity, epidemiology, and genetics of class D β-lactamases. Antimicrob. Agents Chemother. 54, 24–38. doi: 10.1128/AAC.01512-08 19721065 PMC2798486

[B28] Pompilio A. Di Bonaventura G. (2026). An unexpected inverse relationship between biofilm formation and antibiotic resistance in Stenotrophomonas maltophilia. Antibiot (Basel Switzerland) 15 (1), 85. doi: 10.3390/antibiotics15010085 41594122 PMC12838282

[B29] Rani K. Sharma A. Sharma S. Sheba P. Raj V. S. (2024). Solithromycin in combination with other antimicrobial agents against the carbapenem resistant Klebsiella pneumoniae (CRKP). Indian J. Microbiol. 64 (2), 540–547. doi: 10.1007/s12088-024-01188-8 39011018 PMC11246330

[B30] Saksena R. Gaind R. Sinha A. Kothari C. Chellani H. Deb M. (2018). High prevalence of fluoroquinolone resistance amongst commensal flora of antibiotic naïve neonates: a study from India. J. Med. Microbiol. 67, 481–488. doi: 10.1099/jmm.0.000686 29458558

[B31] Shahari A. S. Palanisamy N. K. Nor F. M. Shahari A. S. Palanisamy N. K. Nor F. M. (2025). Genetic profiling of multidrug-resistant Acinetobacter baumannii from a tertiary care center in Malaysia. Microbiol. Spectr. 13, e00872–24. doi: 10.1128/spectrum.00872-24 PMC11792510 39704504

[B32] Sharma A. Tripathi S. Rani K. Vibhuti A. Pati R. Samuel P. V. (2025). The antimicrobial spectrum to manage the co-infections of Pseudomonas aeruginosa and Staphylococcus aureus. Discov. Bact. 3, 28. doi: 10.1007/s44351-025-00028-4 30311153

[B33] Sultana Q. Jayanthi R. S. Pavani K. (2025). Isolation and prevalence of multi-drug resistant Acinetobacter from various clinical samples in a tertiary care hospital 15, 83–92. doi: 10.52403/ijhsr.20251211

[B34] Tacconelli E. Carrara E. Savoldi A. Harbarth S. Mendelson M. Monnet D. L. . (2018). Discovery, research, and development of new antibiotics: the WHO priority list of antibiotic-resistant bacteria and tuberculosis. Lancet Infect. Dis. 18, 318–327. doi: 10.1016/S1473-3099(17)30753-3 29276051

[B35] Thacharodi A. Vithlani A. Hassan S. Alqahtani A. Pugazhendhi A. (2024). iScience review carbapenem-resistant Acinetobacter baumannii raises global alarm for new antibiotic regimens. ISCIENCE 27, 111367. doi: 10.1016/j.isci.2024.111367 39650735 PMC11625361

[B36] Uruén C. Chopo-Escuin G. Tommassen J. Mainar-Jaime R. C. Arenas J. (2020). Biofilms as promoters of bacterial antibiotic resistance and tolerance. Antibiot (Basel Switzerland) 10 (1), 3. doi: 10.3390/antibiotics10010003 33374551 PMC7822488

[B37] Wang L. Chen Q. Qin Y. Yi X. Zeng H. (2024). Analysis of carbapenem-resistant Acinetobacter baumannii carbapenemase gene distribution and biofilm formation 15, 1–11. doi: 10.62347/kbsb9946 PMC10944714 38505565

[B38] World Health Organization (WHO) (2024). Global Antimicrobial Resistance and Use Surveillance System (GLASS). (Geneva, Switzerland: WHO Press). Available online at: https://www.who.int/publications/i/item/9789240093461 (Accessed July 16, 2024).

