# Supplementary material for: Carbapenem resistance genes and biofilm−virulence uncoupling in Acinetobacter baumannii
Source: Front Antibiot. 2026 Jul 29;5:1888133. doi: 10.3389/frabi.2026.1888133 (PMC13462743; doi:10.3389/frabi.2026.1888133)
Supplement: Supplementary file 1 [file SupplementaryFile1.docx]

**Supplementary: Table 1**

| **Class** | **Antibiotic** | **Resistant n(%)** | **Intermediate n(%)** | **Susceptible n(%)** |
| --- | --- | --- | --- | --- |
| **Carbapenem** | Meropenem | 121 (60.5) | 3 (1.5) | 76 (38.0) |
|  | Imipenem | 121 (60.5) | 3 (1.5) | 76 (38.0) |
| **Fluoroquinolone** | Ciprofloxacin | 168 (84.0) | 10 (5.0) | 22 (11.0) |
|  | Levofloxacin | 168 (84.0) | 8 (4.0) | 24 (12.0) |
| **Aminoglycoside** | Amikacin | 135 (67.5) | 12 (6.0) | 53 (26.5) |
|  | Gentamicin | 117 (58.5) | 14 (7.0) | 69 (34.5) |
| **3rd-gen Cephalosporin** | Ceftazidime | 149 (74.5) | 9 (4.5) | 42 (21.0) |
|  | Cefotaxime | 151 (75.5) | 7 (3.5) | 42 (21.0) |
|  | Ceftriaxone | 150 (75.0) | 8 (4.0) | 42 (21.0) |
| BL/BLI | Piperacillin-tazobactam | 122 (61.0) | 15 (7.5) | 63 (31.5) |
| Glycylcycline | Tigecycline | 42 (21.0) | 34 (17.0) | 124 (62.0) |
| Polymyxin | Colistin^†^ | 8 (4.0) | 12 (6.0) | 180 (90.0) |

*† Colistin MIC breakpoints interpreted per EUCAST (no CLSI criteria available for Acinetobacter spp.). BL/BLI: β-lactam/β-lactamase inhibitor.*

**Supplementary Table: 2**

| **Gene** | **Primer** | **Sequence (5'→3')** | **Reference** |
| --- | --- | --- | --- |
| *bla*OXA-51-like *(species ID)* | F | TAATGCTTTGATCGGCCTTG | (F. et al., 2006) |
|  | R | TGGATTGCACTTCATCTTGG |  |
| *bla*OXA-23 | F | ATCGGATTGGAGAACCAGA | (Hou & Yang, 2015) |
|  | R | ATTTCTGACCGCATTTCCAT |  |
| *bla*OXA-24 | F | GGTTAGTTGGCCCCCTTAAA | (Hou & Yang, 2015) |
|  | R | AGTTGAGCGAAAAGGGGATT |  |
| *bla*OXA-58 | F | AAGTATTGGGGGCTTGTGCTG | (Hou & Yang, 2015) |
|  | R | CCCCTCTGCGCTCTATAC |  |
| *bla*NDM-1 | F | GGTCGTCATTTCCGGAAAG | (Nasrabadi et al., 2024) |
|  | R | CGGAATGGCTCATCACGATC |  |
| *bla*VIM | F | GATGGTGTTTGGTCGCATA | (Nasrabadi et al., 2024) |
|  | R | CGAATGCGCAGCACCAG |  |
| *bla*IMP | F | GGAATAGAGTGGCTTAAYTCTC | (Nasrabadi et al., 2024) |
|  | R | GGTTTAAYAAAACAACCACC |  |
| *bla*TEM | F | ATGAGTATTCAACATTTCCG | (Shahbazi et al., 2020) |
|  | R | CCAATGCTTAATCAGTGAGG |  |
| AmpC | F | TAAACACCACATATGTTCCG | (Germán & Jesús, 2000) |
|  | R | ACTTACTTCAACTCGCGACG |  |
| *bla*PER-1 | F | GCAACTGCTGCAATACTCGG | (Yang et al., 2019) |
|  | R | ATGTGCGACCACAGTACCAG |  |
| *bap* | F | TGCTGACAGTGACGTAGAACCACA | (Yang et al., 2019) |
|  | R | TGCAACTAGTGGAATAGCAGCCCA |  |
| *ompA* | F | GTTAAAGGCGACGTAGACG | (Yang et al., 2019) |
|  | R | CCAGTGTTATCTGTGTGACC |  |
| *csuE* | F | CATCTTCTATTTCGGTCCC | (Yang et al., 2019) |
|  | R | CGGTCTGAGCATTGGTAA |  |

Abbreviations: bp, base pairs; F, forward primer; R, reverse primer. All PCR reactions were performed in 25 µL volumes using Promega 2× GoTaq Master Mix with initial denaturation at 95°C for 5 min, followed by 30 cycles (94°C for 45 s; gene-specific annealing temperature 50-60°C for 1 min; 72°C for 1 min), and a final extension at 72°C for 10 min. Amplicons were resolved on 1.5% ethidium bromide-stained agarose gels alongside a 100 bp ladder.

**Supplementary Table 3**: Distribution of antimicrobial resistance and virulence genes among *A. baumannii* isolates (N = 200).

| **Gene Type** | **Gene** | **Present n (%)** | **Absent n (%)** |
| --- | --- | --- | --- |
| **AMR Gene** | *bla*OXA-51 (intrinsic) | 200 (100.0) | 0 (0.0) |
|  | *bla*OXA-23 | 122 (61.0) | 78 (39.0) |
|  | AmpC | 158 (79.0) | 42 (21.0) |
|  | *bla*NDM-1 | 5 (2.5) | 195 (97.5) |
|  | *bla*OXA-58 | 4 (2.0) | 196 (98.0) |
|  | *Bla*TEM | 5 (2.5) | 195 (97.5) |
|  | *bla*VIM | 6 (3.0) | 194 (97.0) |
|  | *bla*IMP | 1 (0.5) | 199 (99.5) |
|  | *bla*OXA-24 | 3 (1.5) | 197 (98.5) |
|  | *bla*PER-1 | 121 (60.5) | 79 (39.5) |
| **Virulence Gene** | *Bap* | 151 (75.5) | 49 (24.5) |
|  | *ompA* | 157 (78.5) | 43 (21.5) |
|  | *csuE* * | 125 (62.8) | 74 (37.2) |

** One isolate had missing csuE data (valid N = 199). All other percentages calculated from N = 200. AMR: antimicrobial resistance.*

**Supplementary Figure: 1** Biofilm formation in 24-well plates using microtiter plate (MtP) assay, categorized as strong, moderate, weak formers based on crystal violet staining intensity.

1500 bp

500 bp

200 bp

500 bp

200 bp

**Supplementary Figure: 2**: AMR genes screened in CRABs. AmpC (1243bp), OXA-23 (498bp), TEM (900bp), OXA-51 (353bp), NDM-1 (154bp), VIM (815bp), OXA-58 (599bp), OXA-24 (146bp)

1500 bp

500 bp

200 bp

**Supplementary Figure 3:** PCR amplification of biofilm-associated protein (bap 184bp) and virulence genes beta-lactamase PER-1 (blaPER-1 800bp), outer membrane protein A (ompA 478bp), and chaperone-usher pilus assembly protein E (csuE 168bp) in CRABs. All screened genes were detected in >75% of isolates.
